# Height, Zinc and Soil-Transmitted Helminth Infections in Schoolchildren: A Study in Cuba and Cambodia

**DOI:** 10.3390/nu7043000

**Published:** 2015-04-20

**Authors:** Brechje de Gier, Liliane Mpabanzi, Kim Vereecken, Suzanne D. van der Werff, Patrick C. D’Haese, Marion Fiorentino, Kuong Khov, Marlene Perignon, Chhoun Chamnan, Jacques Berger, Megan E. Parker, Raquel Junco Díaz, Fidel Angel Núñez, Lázara Rojas Rivero, Mariano Bonet Gorbea, Colleen M. Doak, Maiza Campos Ponce, Frank T. Wieringa, Katja Polman

**Affiliations:** 1 Department of Health Sciences, VU University Amsterdam, Amsterdam 1081HV, The Netherlands; E-Mails: s.ruhe@vumc.nl (S.D.W.); c.m.doak@vu.nl (C.M.D.); m.camposponce@vu.nl (M.C.P.); c.b.polman@vu.nl (K.P.); 2 Department of Biomedical Sciences, Institute of Tropical Medicine, Antwerp 2000, Belgium; E-Mails: l.mpabanzi@gmail.com (L.M.); KimVereecken@itg.be (K.V.); 3 Laboratory of Pathophysiology, University of Antwerp, Wilrijk, B-2610, Belgium; E-Mail: Patrick.DHaese@uantwerpen.be; 4 Institut de Recherche pour le Développement, UMR-204 NutriPass IRD-UM-SupAgro, Montpellier 34394, France; E-Mails: marionfiorentino@hotmail.com (M.F.); perignonmarlene@gmail.com (M.P.); jacques.berger@ird.fr (J.B.); franck.wieringa@ird.fr (F.T.W.); 5 Department of Fisheries Post-Harvest Technologies and Quality Control, Fisheries Administration, Phnom Penh 12301, Cambodia; E-Mails: kuong.kh@gmail.com (K.K.); chhounchamnan@gmail.com (C.C.); 6 PATH, Seattle WA 98109, USA; E-Mail: mparker@path.org (M.P.); 7 National Institute of Hygiene, Epidemiology and Microbiology, Havana 10300, Cuba; E-Mails: rjunco@inhem.sld.cu (R.J.D.); mbonet@inhem.sld.cu (M.B.G.); 8 Pedro Kourí Institute of Tropical Medicine, Havana 11400, Cuba; E-Mails: fan@ipk.sld.cu (F.A.N.); LRojas@ipk.sld.cu (L.R.R.)

**Keywords:** zinc, soil-transmitted helminth infections, child, growth, height, Cambodia, Cuba

## Abstract

Soil-transmitted helminth (STH) infections and zinc deficiency are often found in low- and middle-income countries and are both known to affect child growth. However, studies combining data on zinc and STH are lacking. In two studies in schoolchildren in Cuba and Cambodia, we collected data on height, STH infection and zinc concentration in either plasma (Cambodia) or hair (Cuba). We analyzed whether STH and/or zinc were associated with height for age z-scores and whether STH and zinc were associated. In Cuba, STH prevalence was 8.4%; these were mainly *Ascaris lumbricoides* and *Trichuris trichiura* infections. In Cambodia, STH prevalence was 16.8%, mostly caused by hookworm. In Cuban children, STH infection had a strong association with height for age (aB-0.438, *p* = 0.001), while hair zinc was significantly associated with height for age only in STH uninfected children. In Cambodian children, plasma zinc was associated with height for age (aB-0.033, *p* = 0.029), but STH infection was not. Only in Cambodia, STH infection showed an association with zinc concentration (aB-0.233, *p* = 0.051). Factors influencing child growth differ between populations and may depend on prevalences of STH species and zinc deficiency. Further research is needed to elucidate these relationships and their underlying mechanisms.

## 1. Introduction

Height for age, expressed as z-scores of internationally accepted reference curves, is recommended by the World Health Organization (WHO) and the United Nations Children’s Fund (UNICEF) and Food and Agriculture Organization (FAO) as an indicator of chronic undernutrition [1]. Undernutrition can be caused by insufficient intake of macronutrients, micronutrients or both. Poor growth has been associated with insufficient intake and/or absorption of micronutrients [2]. An important micronutrient deficiency prevalent in many middle- and low-income countries is zinc deficiency, for which over 20% of the world’s population is estimated to be at risk [3]. Zinc, a trace metal micronutrient, influences many physiological functions, among which growth [4,5]. Deficiency in zinc is recognized as a major cause of morbidity and mortality in developing countries [6,7]. Though generally accepted as a public health concern, documentation on zinc deficiency at the population level remains challenging, as there is no gold standard for the measurement of zinc levels [8,9]. To date, plasma/serum zinc concentration, dietary intake, and stunting prevalence are the best-known indicators of zinc deficiency [6].

Infections with soil-transmitted helminths (STH) such as *Ascaris lumbricoides*, *Trichuris trichiura* and hookworm affect approximately a quarter of the world’s population, and the vast majority of these populations live in middle- and low-income countries in (sub)tropical regions [10]. STH infections have been associated with reduced height for age and stunting, and are strongly related to poverty [11,12]. Populations of these endemic regions often show a poor nutritional status [13]. Zinc deficiency and STH infections are thus likely to coexist in these areas. Moreover, several studies have suggested a role for zinc in susceptibility to STH infections [14,15]. Although the effects of zinc deficiency and STH infections on growth have both been widely studied, data on the association between zinc, STH infection and growth are scarce.

Poor nutritional status and STH infection are intricately linked, whereby STH infection can lead to malnutrition and malnutrition may increase susceptibility to STH infection [15]. Likewise, STH infections and poor nutritional status can affect growth, either independently or in combination. Economic development, population nutritional status, as well as STH species distributions vary greatly between STH endemic countries. For example, Cambodia remains a low-income country with a high prevalence of stunting despite considerable economic development and significant improvement in its population health conditions since the end of the civil war. Food insecurity is still a reality for many of its inhabitants, and, additionally, a high prevalence of STH infection has been reported, mostly by hookworm and *A. lumbricoides* [16]. In contrast, Cuba, which is also an STH endemic country, has a high development index and is categorized as an upper middle-income country. In Cuba, the epidemiological transition has firmly settled in and overweight rather than underweight is currently a public health concern [17]. Estimates of zinc deficiency prevalence are not available for these countries. The present paper aimed at assessing the associations between height for age, zinc status and STH infections in school-aged children in these two different populations.

## 2. Methods

### 2.1. Study Population Cuba

A cross-sectional study within school-aged children was performed in 2009 in San Juan y Martínez, Pinar del Rio, a municipality in the West of Cuba. The municipality is situated in a rural mountainous area, which is endemic for STHs [18]. From 13 randomly selected schools, 1389 children were included in the study. Written informed consent was obtained from the parents or caretakers of each child. The study was approved by the ethical committees of the Institute of Tropical Medicine in Antwerp (Belgium), the Pedro Kourí Institute of Tropical Medicine and the National Institute for Hygiene, Epidemiology and Microbiology in Havana (Cuba).

### 2.2. Study Population Cambodia

Data from the baseline measurements of a randomized controlled trial on the effects of multiple-micronutrient-fortified rice on child nutrition and morbidity were used. The trial was conducted in rural Kampong Speu province, Cambodia, in November 2012. Children from 20 randomly selected schools were included (*N* = 2471). All parents or caretakers were asked to sign an informed consent form. Ethical approval was obtained from the Cambodian Ministry of Health, Education and Planning and the Ethical Review board of PATH, USA.

### 2.3. Height for Age

Height measurements were performed to the nearest 0.1 cm by trained investigators using standard procedures. Age in months was calculated from the children’s birth date, retrieved via interviews and verified by school records and birth certificates (Cambodia). Height for age z-scores were calculated according to the WHO 2007 reference curves, using the WHO macro for SPSS [19]. Stunting was defined as height for age z-score below −2 SD. For analyses where age or height for age as continuous covariates were not linearly associated with the dependent variable, data were categorized. Cutoffs were chosen so that three categories of approximately equal group size were made. Because age and height for age ranges differed between both populations, the categories were defined differently per population. In the Cuban data, age was categorized as 4 to 7, ≥7 to 10 and ≥10 to 13 years old. Cuban height for age z-scores were categorized as <0, 0–1 and >1 SD. In the Cambodian data, age was categorized as 5 to 10, ≥10 to 13 and ≥13 to 17 years old. Here, height for age z-scores were categorized as ≤−2, −2 to 0 and >0 SD.

### 2.4. Parasitology and Treatment

In both countries, one fresh stool sample was collected from each child. Stools were examined by the Kato-Katz technique (duplicate 25 mg smears) according to standard procedures to detect *A. lumbricoides*, *T. trichiura*, and/or hookworm [20]. Infection intensity was recorded as eggs per gram feces (epg) and classified according to WHO guidelines. STH positive children received anthelminthic treatment: in Cuba, one single dose of 500 mg mebendazole, which has been evaluated and is the treatment of choice in Cuba [21] and in Cambodia, one single dose of 400 mg albendazole was given [22].

### 2.5. Plasma Zinc and Inflammation

In Cambodia, zinc was measured in plasma. C-reactive protein (CRP) and alpha-1 acid glycoprotein (AGP) were measured alongside plasma zinc, in order to adjust for the effects of inflammation on plasma zinc concentrations. Plasma zinc and CRP and AGP were measured in 5 mL of venous blood, obtained from participants by venipuncture. Plasma zinc concentration was measured by flame atomic absorption spectrophotometry and verified against reference material at the National Institute for Nutrition in Hanoi, Vietnam. Deficiency was defined as plasma zinc below 9.9 μmol L^−1^ for children below the age of 10, below 10.1 μmol L^−1^ for girls age 10 and older and plasma zinc below 10.7 μmol L^−1^ for boys age 10 and older [6]. In 100 μL plasma aliquots, CRP and AGP were measured by sandwich enzyme-linked immunosorbent (ELISA) techniques (VitMin Laboratories, Germany) [23]. Inflammation categories were defined as elevated CRP only, elevated AGP only, both CRP and AGP elevated or no elevated CRP or AGP. Elevated CRP was defined as values above 5 mg L^−1^, elevated AGP was defined as >1 g L^−1^ [24].

### 2.6. Hair Zinc

In Cuba, zinc was measured in hair. Two months before the measurements, parents or guardians of the participating children were asked not to cut the hair of their children. Approximately 200–500 mg of hair was collected with the use of stainless steel scissors in the nape or (lower) occipital region of the head approximately 1.5 cm away the scalp. The distal ends of the hair were cut from the samples, leaving a specimen of approximately 2 cm in length. Samples were stored in plastic bags at −20 °C until the determination of the zinc content. In the laboratory, the samples were analyzed for zinc content by spectrometry. In order to assure the quality of the zinc measurements taken, samples first underwent a washing procedure, to remove exogenous zinc without removing endogenous zinc. Ultra-pure reagents and pretested vials were used. Zinc analysis was done according to the protocol of D’Haese *et al*. [25]. A cutoff value of 70 μg g^−1^ wet weight was used to define zinc deficiency [6,26]. Due to funding restraints, hair zinc was measured in a subset of 230 Cuban children.

### 2.7. Statistical Analysis

Analyses were done using SPSS software version 21 (IBM, NY, USA). Hair zinc followed a skewed distribution, therefore the data for this variable were natural log-transformed for regression analysis and expressed as median and interquartile range for descriptive analysis. The variable STH infection refers to the presence of any STH infection, ‘zinc’ refers to zinc concentration and ‘height for age’ refers to height for age z-score in all analyses. For statistical testing, linear regression analysis was performed with height for age z-scores, plasma zinc or the natural logarithm of hair zinc as continuous dependent variables. Covariates of each analysis are specified in the table footnotes. In the analyses of associations between zinc and STH infection with height for age, age was included as a continuous covariate and inflammation categories were included as categorical covariate for the plasma zinc data. In the analysis of associations between zinc and STH infection, covariates age and height for age z-scores were included as categorical variables, created from age and height for age categories. Sex was added as binary covariate in all analyses. Statistical significance was defined as a *p* value below 0.05, for variables as well as interaction terms.

## 3. Results

### 3.1. Characteristics of the Study Populations

The mean height for age z-score (0.06) of the Cuban children was significantly higher than the median of the reference population (z-score = 0) (*p* = 0.03). Only 21 (1.6%) of the Cuban children presented with stunting (Table 1). In the Cambodian children, mean height for age z-scores were significantly lower than 0 (*p* < 0.001) and stunting was common (42.9%). Zinc deficiency was highly prevalent in Cambodia (92.8%), whereas zinc deficiency was found in only 12.2% of the Cuban children. Prevalence of STH infections was 8.4% and 16.8% for Cuba and Cambodia, respectively. In the Cuban study, the most common STH infections were *A. lumbricoides* (61.4%) and *T. trichiura* (36.8%), while hookworm (97.0%) was the predominant STH infection in Cambodia. In both populations, most STH infections were of light intensity (Table 1).

**Table 1:** Characteristics of the study populations.

	Cuba (*N* = 1389)	Cambodia (*N* = 2471)
	*n* (%) or mean ± sd	*n* (%) or mean ± sd
Age (years)	8.14 ± 2.07	9.68 ± 2.27
Sex (female)	640 (47.0%)	1236 (50.0%)
Height for age z-score	0.06 ± 1.04	−1.81 ± 1.05
Stunted	21 (1.6%)	1056 (42.9%)
STH infection ^a^	114 (8.4%)	302 (16.8%)
*Ascaris lumbricoides*	70 (5.2%)	5 (0.3%)
Light (<5.000 epg)	55 (4.1%)	5 (0.3%)
Moderate (5.000–50.000 epg)	15 (1.1%)	0
Heavy (>50.000 epg)	0	0
*Trichuris trichiura*	42 (3.1%)	6 (0.3%)
Light (<1.000 epg)	38 (2.8%)	6 (0.3%)
Moderate (1.000–10.000 epg)	2 (0.1%)	0
Heavy (>10.000 epg)	2 (0.1%)	0
Hookworm	15 (1.1%)	293 (16.3%)
Light (<2.000 epg)	13 (1.0%)	283 (15.8%)
Moderate (2.000–4.000 epg)	0	9 (0.5%)
Heavy (>4.000 epg)	2 (0.1%)	1 (0.1%)
Hair zinc (μg g^−1^)	113 (91–137) ^b^	n.a.
Zinc deficiency ^c^	28 (12.2%)	n.a.
Plasma zinc ^d^ (μmol L^−1^)	n.a.	7.65 ± 1.69
Zinc deficiency ^e^	n.a.	1884 (92.8%)
Inflammation		
No inflammation	n.a.	1450 (60.5%)
Only CRP elevated	n.a.	8 (0.3%)
Only AGP elevated	n.a.	816 (34.1%)
CRP & AGP elevated	n.a.	122 (5.1%)

^a^: *N*= 1353 (Cuba) or *N* = 1795 (Cambodia); ^b^: median (IQR), *N* =230; ^c^: hair zinc < 70 μg g^−1^; ^d^: *N* =2112; ^e^: age 4–9: plasma zinc < 9.9 μmol L^−1^; girls age 10 and up: plasma zinc < 10.1 μmol L^−1^ boys age 10 and up: plasma zinc < 10.7 μmol L^−1^, *N* = 2030; STH: soil-transmitted helminth; epg: eggs per gram feces; n.a.: not applicable

### 3.2. Associations between Height for Age, Zinc and STH Infection

STH infected Cuban children had on average lower height for age compared to their uninfected peers (Table 2), and regression analysis showed a significant negative association between STH infection and height for age (Table 3). The association between hair zinc and height for age was not significant but did show a positive trend. In Cambodia, plasma zinc, but not STH infection, was significantly associated with height for age (Table 3). In both populations, STH x zinc interaction terms were not statistically significant. However, when stratifying for STH infection, in the uninfected Cuban children a significant, positive association (aB-0.471, *p* = 0.033) was found between hair zinc and height for age.

**Table 2:** Zinc and height for age in STH infected and uninfected children.

		*N*	Zinc concentration	*N*	Height for age z score (mean ± sd)
Cuba	STH uninfected	160	112.55 (88.3–136.0) ^a^	1251	0.11 ± 0.97
	STH infected	70	113.35 (94.4–143.7) ^a^	117	−0.31 ± 1.16
Cambodia	STH uninfected	1239	7.74 ± 1.70 ^b^	1450	−1.81 ± 1.05
	STH infected	254	7.52 ± 1.70 ^b^	296	−1.84 ± 1.09

^a^: Hair zinc in μg g^−1^, median (IQR); ^b^: Plasma zinc in μmol L^−1^, mean ± sd.; STH: soil-transmitted helminth

In the Cuban study, the median hair zinc concentration was slightly higher in STH infected than in uninfected children (Table 2), but the result of the regression analysis was not statistically significant (Table 4). In contrast, STH infected children in the Cambodian study had on average lower plasma zinc concentrations than their uninfected peers (Table 2). This association was borderline significant (Table 4).

**Table 3:** Linear regression models of height for age by STH infection and zinc.

	independent variable	*N*	aB ^a^	*p*
Cuba ^b^	STH infection	226	−0.483	0.001
	Zinc		0.335	0.082
Cambodia ^c^	STH infection	1448	−0.008	0.902
	Zinc		0.033	0.029

^a^: regression coefficient; ^b^: adjusted for sex and age in months; ^c^: adjusted for sex, age in months and inflammation categories; STH: soil-transmitted helminth

**Table 4:** Linear regression models of zinc by STH infection.

	Variable	N	aB	*p* value
Cuba ^a^	STH infection	230	0.068	0.206
Cambodia ^b^	STH infection	1795	−0.233	0.051

^a^: adjusted for sex, age categories and height for age categories; ^b^: adjusted for inflammation categories, sex, age categories and height for age categories; STH: soil-transmitted helminth

## 4. Discussion

The present study showed different associations between height for age, STH infection and zinc in Cuban and Cambodian schoolchildren. In the Cuban study population STH infection was significantly associated with lower height for age, while hair zinc concentrations were not. Conversely, in the Cambodian study population plasma zinc, but not STH infection, was significantly associated with higher height for age.

The two populations were markedly different in mean height for age. The Cuban schoolchildren were on average taller than the reference population [20] and stunting was rare. These characteristics generally indicate an adequate zinc status at population level [6] and this was confirmed by the observed hair zinc values. STH infection appeared to have a stronger effect than zinc on height for age in Cuban children. Because stunting was rare in the Cuban study population, the associations occurred in children of normal height. The Cambodian schoolchildren included in the study had a low mean height for age compared to the reference population [20] and stunting was common. The observed stunting suggested a zinc deficient population [6], which was indeed corroborated by the observed plasma zinc values. In these children, STH infection was not associated with height for age.

This study also examined the relation between zinc and STH infection. Plasma zinc concentrations were lower in STH infected Cambodian children than in their uninfected peers. This association was borderline significant. Few other studies have addressed associations between zinc and STH infection. In 2009, Rosado *et al*. found that while zinc supplementation increased height for age in Mexican infants, this effect was diminished by *Ascaris* infection [27]. Kongsbak *et al*. found *T. trichiura* to be a significant predictor of serum zinc in a Bangladeshi population where stunting was common [14]. In this study, *T. trichiura* had a larger effect on serum zinc than did *A. lumbricoides*, suggesting species-specific differences. Osei *et al*. did not find serum zinc to differ significantly between STH infected and uninfected Indian children [28]. Two recent meta-analyses found no significant effect of zinc supplementation on STH (re-) infection rate [29,30]. The present study did not distinguish between the effects of the different STH species. In our Cambodian study, children carried almost exclusively hookworm infections. Hence, STH species-specific effects on zinc could not be determined in this population. Likewise, a comparison between zinc deficient and zinc sufficient children in STH infection was not possible, since almost all of the Cambodian children were zinc deficient.

The different associations between STH and stunting found in the two populations might reflect the difference in predominating STH species. In the present study, the Cuban children were more often infected with *A. lumbricoides* or *T. trichiura*, while hookworm was the prevailing STH infection in Cambodia. These species have distinct life cycles and might therefore have quite different effects on nutritional status [13,31]. Recently, in a study conducted in children in the Philippines, Papier *et al*. showed that the proportion of stunted children was significantly higher among children infected with hookworm than among children infected with *A. lumbricoides*, and *T. trichiura* [32]. These findings are corroborated by the results of this study.

This study has some limitations, warranting caution in its interpretation. Since the present study is cross-sectional, causality cannot be inferred. STH infections and zinc deficiency are often put forward as important causes of child stunting [6,13]. However, reduced height for age might also reflect a generally poor nutritional status, which can influence both zinc uptake and susceptibility to infections. Stunting is also strongly related to poverty, as are STH infections and zinc status [12,33]. Moreover, observed associations between height, zinc and STH might all be explained in the context of ‘environmental enteropathy’; repeated exposure to intestinal pathogens resulting in inflammation and remodeling of the mucosa, causing widespread malabsorption [34].

Associations between zinc and helminths can also be interpreted in various ways. STH infection might damage or block the intestinal mucosa, resulting in reduced uptake of nutrients [13]. Additionally, the STH might compete with the host for essential elements. Inflammation resulting from infection can also lead to reduced micronutrient levels in plasma, induced by the acute phase response [35]. For this reason, inflammation was taken into account in the present analysis. On the other hand, zinc status can influence susceptibility to infection by its effects on immune function [6].

While the importance of assessing zinc levels has been recognized for many years, a reliable and representative method to measure zinc remains a challenge. Serum or plasma zinc is considered the best available biomarker of zinc deficiency in populations [6]. It has been shown that plasma zinc reflects dietary zinc intake and that it responds consistently to zinc supplementation [6,36]. However, the timing of blood collection and fasting status influence the zinc concentrations measured in plasma [37]. Moreover, zinc is considered a ‘type-II’ nutrient, meaning that no real stores exist, and that growth faltering is one of the key features of deficiency [38]. Associations between low zinc concentration in hair and poor growth have been documented [6]. Hair zinc has been shown to increase after supplementation [37]. However, it has been argued that zinc in hair reflects a more extended period of exposure than plasma zinc [6]. It cannot be excluded that differences observed in the present study might be (partly) due to the use of different methods of zinc measurement. Presently, there are no reliable data on the correlation between hair zinc values and plasma or serum zinc values. Moreover, although the effects of the acute phase response on plasma zinc levels are widely recognized, there is currently no standard method of accounting for this in school-age children [24,39].

## 5. Conclusion

Based on the results of this study, we recommend that STH infection and zinc status at population level should be taken into account when assessing the potential factors contributing to stunting. It is essential to define a standard and reliable method of measuring zinc and accounting for inflammation effects in order to further elucidate associations between zinc, STH infection and growth. In populations living in STH endemic areas, a possible association between zinc and STH should be considered. This will improve (the evidence base for) interventions on child growth, for instance by pairing zinc supplementation with helminth control strategies.
